# Facilitators of research registry enrollment and potential variation by race and gender

**DOI:** 10.1017/cts.2018.326

**Published:** 2018-11-27

**Authors:** Crystal M. Glover, Christina Creel-Bulos, Lisa M. Patel, Scarlett Ellis During, Karen L. Graham, Yadira Montoya, Susan Frick, Judy Phillips, Raj C. Shah

**Affiliations:** 1 Department of Behavioral Sciences, Rush University Medical Center, Chicago, IL, USA; 2 Rush Alzheimer’s Disease Center, Chicago, IL, USA; 3 Department of Emergency Medicine, Barnes Jewish Hospital/Washington University, St. Louis, MI, USA; 4 Department of Internal Medicine, Rush University Medical Center, Chicago, IL, USA; 5 Alexian Brothers Medical Center, Neurosciences Clinical Research, Elk Grove Village, IL, USA; 6 Department of Family Medicine, Rush University Medical Center, Chicago, IL, USA

**Keywords:** Research registries, older adults, cognitive health, health equity

## Abstract

**Introduction:**

Little is known about what motivates people to enroll in research registries. The purpose of this study is to identify facilitators of registry enrollment among diverse older adults.

**Methods:**

Participants completed an 18-item Research Interest Assessment Tool. We used logistic regression analyses to examine responses across participants and by race and gender.

**Results:**

Participants (N=374) were 58% black, 76% women, with a mean age of 68.2 years. All participants were motivated to maintain their memory while aging. Facilitators of registry enrolled varied by both race and gender. Notably, blacks (estimate=0.71, *p*<0.0001) and women (estimate=0.32, *p*=0.03) were more willing to enroll in the registry due to home visits compared with whites and men, respectively.

**Conclusions:**

Researchers must consider participant desire for maintaining memory while aging and home visits when designing culturally tailored registries.

## Introduction

Participant recruitment into research studies and subsequent retention have continued to pose a challenge to investigators [1–4]. Issues related to recruitment and retention have been wide-reaching with impacts on both survey and observational research, as well as randomized controlled trials and clinical trials [5]. Investigators oftentimes have failed to enroll target numbers of participants into research studies; with some unable to enroll any participants [3,6,7]. Low rates of research participation directly implicate a study’s inability to reach its recruitment targets. Therefore, a study faces unanticipated extensions of study timeframes, threats to internal and external validity, and the delayed or complete absence of progress related to interventions, education, and drug development.

While lower rates of research participation among all Americans remain a concern, a lack of diversity among research participants also presents a problem to study recruitment. The Eliminating Disparities in Clinical Trials initiative performed a critical analysis of research participation and noted the need for the inclusion of vulnerable populations including racial/ethnic minorities and older adults (defined as age 65 years and older) [8]. Although racial/ethnic minorities represent 30% of the US population, they have only comprised 17% of clinical trial participants included in Food and Drug Administration New Drug Application packets [9]. Similarly, in 2014, older adults comprised 14.5% of the US population [10] and carried 60% of the national disease burden but only represented 32% of phases II and III clinical trial participants [11]. In addition, many older adults also belong to racial/ethnic minority groups [3] with older minorities anticipated to constitute 28.5% of all older adults in 2030—an expected increase from 21.2% in 2013 [12].The generalizability and efficacy of research findings rely on the recruitment of diverse participants into research studies [13,14].

Various factors serve as facilitators of research participation. Altruism—helping family and the community—represents one such factor. Others include research participation as convenient, low risk, and personally beneficial such as the receipt of financial compensation and disease/condition improvement. Conversely, several factors do not enable research participation, including mistrust of research and related systems, competing life demands such as family obligations or transportation needs, potential exposure of personal health-related information, a lack of access to information about research studies, and US immigration status (i.e., legal or undocumented) [13,15,16]. Unique factors may also be specific to subgroups of people. For racial/ethnic minorities, matching between participants and research staff and deployment of cultural competency techniques by research staff serve as facilitators [13,17]. However, older adults may not be motivated to participate in research due to limited mobility, poor communication, and strict study inclusion criteria [18–20].

Research registries represent an emergent methodology for enhancing recruitment and retention efforts [21]. Registries consist of a longitudinal database containing basic demographic characteristics and health information regarding potential research participants. Before the study enrollment period, researchers can screen registries to communicate research opportunities and related study information to eligible, willing, and diverse potential participants. Studies built on high-quality registries can significantly reduce research costs and time [18] and facilitate the recruitment of racial/ethnic minorities into research studies [22]. To our knowledge, few studies have examined what motivates people to enroll in registries [23,24]. The purpose of this study is to understand factors that facilitate enrollment in a registry among community-dwelling older adults who have consented to registry participation. This study also aims to assess potential variation in facilitators by race and gender.

## Materials and Methods

### Participants

We recruited people into a registry for primarily clinical and epidemiological studies focused on healthy aging and aging-related issues. This registry is approved by the Institutional Review Board at Rush University Medical Center (L99032481). Recruitment began during community events (e.g., talks and health fairs) and catered to older adults by discussing topics centered on healthy aging (e.g., diet, exercise, and social activity) and concerns while aging such as cognition. After each event, trained staff asked individuals to (1) enroll in the registry by providing their voluntary verbal informed consent; and (2) answer questions about their decision-making process regarding registry enrollment. The study period was from January 1, 2013 to December 31, 2014.

## Demographic Characteristics

All participants reported their race (i.e., African American/black or white) based on categories from the US Census Bureau (www.census.gov/population/www/socdemo/race.html), as well as gender (i.e., male or female), date of birth, and years of education.

## Research Interest Assessment Tool

The Research Interest Assessment Tool (referred to as “Tool” going forward) was based on the Clinical Research Involvement Scales [25] and the review of previous literature. Clinical Research Involvement Scales questions were modified to reflect specific interests of older adults in conjunction with staff input. The Tool consisted of 18 items using dichotomous “yes” or “no” response options. Questions assessed individual (e.g., “It is important for me to maintain my memory as I age.”), interpersonal (e.g., “My doctor supports/would support my decision to participate in this study.”), and community-level (e.g., “My community is concerned with maintaining memory and thinking skills.”) factors related to decision-making when considering registry enrollment and subsequent research participation. Tool questions referencing “this study” directly pertained to the registry (Table 1).

**Table 1:** Research Interest Assessment Tool

Items
(1) It is important for me to maintain my memory as I age
(2) I, someone I know, or my community will benefit from this trial
(3) I would benefit from the medical care and testing associated with this study
(4) It is too much of a time commitment to become involved in this study
(5) The information about this study was easy to understand
(6) I trust research and research findings
(7) I have had a good experience with research or know someone who has had a good experience with research
(8) I feel empowered and experience a sense of belonging by being involved with this study
(9) Home visits made me more willing to participate in this study
(10) I was motivated and inspired to participate in research studies during the RADC presentation/health fair I attended
(11) My doctor supports/would support my decision to participate in this study
(12) My family supports/would support my decision to participate in this study
(13) I can inspire others to act by participating in this study
(14) I normally do what others expect of me
(15) I was contacted in a timely manner regarding my interest in participating in a study
(16) I appreciated being contacted by the presenter/examiner I met at the presentation/fair I attended
(17) My community is concerned with maintaining memory and thinking skills
(18) My involvement in this study was influenced by my relationship with the Memory Clinic

RADC, Rush Alzheimer's Disease Center.

## Analysis

We used χ^2^ analyses to assess demographic differences between participants who completed the Tool compared with those who did not. We then performed three sets of analyses. First, we obtained descriptive statistics (frequency counts and percentages) for each Tool item for the overall sample, as well as by race and gender. Second, we performed a logistic regression analysis for each Tool item to examine main effects of race and gender. Lastly, we performed separate logistic regression analyses to examine the potential interactive effect of race and gender on each Tool item. All logistic regression models included terms for race, gender, age, and education. For all analyses, a *p*-value of ≤ 0.05 indicated statistical significance. We used SAS^®^, Version 9.4 (SAS Institute Inc., Cary, NC, USA) for all analyses.

## Results

### Participant Characteristics

Of 453 people who enrolled in the registry, 374 agreed to participate in the Tool. Participants who completed the Tool were 58% black, 76% women, with a mean age of 68.2 years, and 14.8 mean years of education. Participants (n=79) who did not complete the Tool were 72% black, 72% women, with a mean age of 66.4 years, and 13.1 mean years of education. Compared with participants who completed the Tool, those who did not were more likely to be black and had fewer years of education. Participants who completed the Tool did not differ by gender and age from those who did not consent to Tool participation (Table 2).

**Table 2:** Demographic characteristics by Tool consent

	Black	Women	Age	Years of education
Consented to Tool (n=374, 83%)	58% (n=215)	76% (n=283)	68.2 (SD=10.8)	14.8 (SD=3.5)
Did not consent to Tool (n=79, 17%)	72% (n=57)	72% (n=57)	66.4 (SD=13.9)	13.1 (SD=4.5)
χ^2^ analyses	χ^2^=8.7, *p*<0.003	NS	NS	χ^2^=15.5, *p*<0.0001

## Tool Responses

### All Participants

A consensus (a rating of 90% or greater) existed across all participants regarding specific Tool items. These items represented key issues for all participants regarding registry participation. All participants indicated it is important for them to maintain their memory as they age. Ninety percent or more of participants reported: (1) they were contacted in a timely manner by research staff; (2) study information was easy to understand; (3) their family supports or would support their decision to participate in this research; (4) they, someone they know, or their community would benefit from this study; (5) they would benefit from medical care and testing associated with this study; and (6) their community is concerned with maintaining memory and thinking skills. Ten percent or less of all participants indicated their study involvement was influenced by their relationship with an affiliated clinic and it was too much of a time commitment to be involved in this study. More than half of all participants reported: (1) their doctor supports or would support their decision to participate in this study; (2) they could inspire others to act by participating in this study; (3) trust in research and research findings; (4) feeling empowered and experiencing a sense of belonging by being involved with this study; (5) normally doing what others expected of them; (6) being motivated and inspired to participate in research studies during a community event; (7) appreciation for being contacted by research staff; (8) being more willing to participate in this study due to home visits; and (9) they had or knew someone who had good experiences with research (Table 3).

**Table 3:** Percentages for each item for overall sample and race and gender groups; and logistic regression analyses for main effects of race and gender

	Percentage for yes response, parameter estimate, *p*-value		
Items	Overall sample	Blacks–whites	Women–men
It is important for me to maintain my memory as I age	100	100 vs.100 NS	100 vs. 100 NS
I, someone I know, or my community will benefit from this trial	90	92 vs. 90 NS	89 vs. 97 NS
I would benefit from the medical care and testing associated with this study	90	95 vs. 84 NS	90 vs. 92 NS
It is too much of a time commitment to become involved in this study	9	9 vs. 6 NS	9 vs. 8 NS
The information about this study was easy to understand	94	97 vs. 90 NS	92 vs. 99 NS
I trust research and research findings	86	81 vs. 92 NS	87 vs. 81 estimate=0.78, *p*=0.02
I have had a good experience with research or know someone who has had a good experience with research	62	60 vs. 65 NS	61 vs. 67 NS
I feel empowered and experience a sense of belonging by being involved with this study	81	81 vs. 82 NS	80 vs. 84 NS
Home visits made me more willing to participate in this study	64	74 vs. 53 estimate=0.71, *p*<0.0001	67 vs. 56 estimate=0.32, *p*=0.03
I was motivated and inspired to participate in research studies during the presentation/health fair I attended	67	80 vs. 45 estimate=0.85, *p*<0.0001	68 vs. 62 NS
My doctor supports/would support my decision to participate in this study	87	84 vs. 93 NS	88 vs. 87 NS
My family supports/would support my decision to participate in this study	92	92 vs. 95 NS	92 vs. 92 NS
I can inspire others to act by participating in this study	87	93 vs. 80 NS	87 vs. 89 NS
I normally do what others expect of me	72	70 vs. 73 NS	73 vs. 68 NS
I was contacted in a timely manner regarding my interest in participating in a study	96	95 vs. 99 NS	98 vs. 92 NS
I appreciated being contacted by the presenter/examiner I met at the presentation/fair I attended	64	81 vs. 38 estimate=1.18, *p*<0.0001	66 vs. 59 NS
My community is concerned with maintaining memory and thinking skills	90	89 vs. 91 NS	88 vs. 94 NS
My involvement in this study was influenced by my relationship with the Memory Clinic	10	11 vs. 10 NS	10 vs. 9 NS

### Racial Differences in Tool Responses

Analyses indicated main effects for race. During community events, blacks reported experiencing more motivation and inspiration to participate in research compared with whites. After community events, blacks expressed more appreciation for being contacted by research staff compared with whites. Blacks also reported that home visits made them more willing to participate in the study compared with whites (Table 3).

### Gender Differences in Tool Responses

Analyses also showed main effects for gender. Women reported more trust in research and research findings compared with men. Women also reported home visits made them more willing to participate in the study compared with men (Table 3).

### Interaction Effects and Tool Responses

An interaction between race and gender did not significantly impact any Tool item.

## Discussion

The current study aims to add to a burgeoning body of literature identifying facilitators of registry enrollment among diverse, community-dwelling older adults. All participants indicated that maintaining their memory as they age was important to them. Results also suggested racial and gender differences. Notably, older blacks and older women reported being more willing to participate in the study due to home visits compared with older whites and older men, respectively. In addition, older blacks experienced more motivation and inspiration to participate in research and appreciated staff contact more in comparison to older whites. Older women also trusted research and research findings more than older men. Race and gender did not interact to impact Tool responses. We have not shared current study results with study participants.

To our knowledge, research remains limited regarding motivators and factors considered in relation to registry enrollment among diverse older adults [14,24]. A larger amount of literature has focused on the development of registries [26], privacy and practical issues associated with registries [27,28], and retention of registry participants and other health outcomes [14,27]. In a smaller body of research, registries have been deemed effective in enhancing minority recruitment efforts [29] and have continued to expand to facilitate the recruitment of older adults [24] especially focused on aging-related health concerns such as dementia [30]. For example, Jefferson *et al*. [22] found that home-based visits and altruism served as facilitators of registry enrollment among older adults.

Current study results lend support to previous research by Jefferson *et al*. [22]. Older adults, especially older blacks and older women, reported home visits as a facilitator of registry participation. Relatedly, older blacks in the current study were more likely to appreciate contact by study staff compared with older whites. Perhaps older minorities, particularly older blacks and older women, prefer to participate in home-based, not just community-based, research opportunities coupled with frequent communication from study staff. Older adults in the current study also reported that they, someone they know, or their community would benefit from research stemming from the registry. Previous research [31,32] has suggested that study advertisements and recruitment materials should highlight the altruistic aspects of research participation. Perhaps registry enrollment and subsequent research participation may provide a mechanism for older adults to give back to society and others.

Conversely, current study results do not fit in with the well-established finding that whites trust research and related findings more than other racial/ethnic groups [13,33,34]. Our results showed no significant difference between older blacks and older whites in terms of trust. Perhaps, as George *et al*. [13] have suggested, the altruistic needs met by research participation may outweigh notions of mistrust among older blacks. We did find that that older women trusted research and related findings more than older men. To our knowledge, previous studies have examined gender differences in research participation but not motivators of registry enrollment. Hence, future research may seek to understand potential gender differences among older adults regarding registry enrollment. Relatedly, current study findings suggested older blacks were more motivated and inspired to participate in research compared with older whites. This finding does not fit with those from a recent systematic review [13] regarding barriers and facilitators of research participation among minorities. However, previous and current findings may differ due to our exclusive focus on older blacks, not black adults at large.

This study has important limitations. First, participants came from a volunteer cohort in the Midwest, and tended to be healthier and more highly educated than the average older adult. Hence, our findings may limit generalizability to older adult populations in the United States and should be replicated in a population-based sample. Our continued community partnerships with organizations serving people of varied socioeconomic status including years of education may produce an even more diverse group of potential registry participants. This study also had a number of strengths including a longitudinal, well-characterized cohort of diverse older adults and a substantial sample of older blacks and older women to address racial and gender differences in facilitators of research registry enrollment.

Overall, more research is needed to understand motivators of registry enrollment, especially potential differences between older blacks and older whites. Understanding what motivates older adults and how facilitators may differ according to race and gender may lead to more culturally competent registry development, recruitment, retention, and research-related materials.
